# TREM2 dependent and independent functions of microglia in Alzheimer’s disease

**DOI:** 10.1186/s13024-022-00588-y

**Published:** 2022-12-23

**Authors:** Jinchao Hou, Yun Chen, Gary Grajales-Reyes, Marco Colonna

**Affiliations:** 1 Department of Pathology and Immunology, Washington University School of Medicine, St. Louis, MO 63110 USA; 2 Department of Neurology, Washington University School of Medicine, St. Louis, MO 63110 USA

**Keywords:** Alzheimer’s disease, Beta-amyloid pathology, Tauopathy, Demyelination, Microglia, TREM2, APOE

## Abstract

Microglia are central players in brain innate immunity and have been the subject of extensive research in Alzheimer’s disease (AD). In this review, we aim to summarize the genetic and functional discoveries that have advanced our understanding of microglia reactivity to AD pathology. Given the heightened AD risk posed by rare variants of the microglial triggering receptor expressed on myeloid cells 2 (TREM2), we will focus on the studies addressing the impact of this receptor on microglia responses to amyloid plaques, tauopathy and demyelination pathologies in mouse and human. Finally, we will discuss the implications of recent discoveries on microglia and TREM2 biology on potential therapeutic strategies for AD.

## Introduction

Alzheimer’s disease (AD) is an age-related neurodegenerative disease which is the most common form of dementia. Pathologically, it is characterized by extracellular beta-amyloid (Aβ) aggregates, intracellular tau neurofibrillary tangles (NFT) and neuronal cell death that ultimately results in cognitive impairment [1]. A wealth of biomarker and imaging studies have led scientists to postulate that Aβ initiates a pathological cascade that results in tau propagation throughout the brain, which correlates with neuronal loss and cognitive impairment [2]. This postulate, referred to as the “amyloid cascade hypothesis” has been a predominant focus for AD research over the past 30 years [3]. The greatest risk factors for AD are age, female sex, and the APOE4 allele [4]. AD mainly affects people over 65: above this age, the risk of developing AD doubles about every five years. The female prevalence of AD is generally attributed to the greater life span of women relative to men [5]. The Apolipoprotein E (APOE) is the major carrier of cholesterol and other types of lipids in the brain [6]. Three major allelic variants of APOE, APOE2, APOE3 and APOE4, have been identified in the population; having at least one APOE4 allele increases the risk of developing AD two- to threefold, while two APOE4 alleles increase the risk to approximate eight- to 12-fold. The reason why APOE4 increases AD risk is not well understood: it is possible that impaired ability of brain cells to process lipids may play a key role in AD.

It has become increasingly clear that the amyloid cascade hypothesis is not sufficient to explain the accumulation of aggregated Aβ and tau in AD. In fact, the progression of AD may also depend on impaired homeostasis of brain cells [7]. Microglia, the principal brain-resident immune cells, have emerged as the initial responders to brain damage, and have been the subject of extensive research in AD [8]. Large-scale genome-wide association studies (GWAS), whole genome/exon sequencing and gene expression network analysis identified more than 40 loci that influence risk for late-onset AD (LOAD, onset age > 65 years) either by altering amino acid sequence (within coding regions) or mRNA transcript levels (within regulatory regions, such as enhancers and promoters) [9, 10]. Of these risk loci, a large number of common and rare risk variants encode either microglial-enriched or microglial-specific molecules, including APOE, TREM2, CD33, INPP5D/SHIP1, SPI1 and ABI3 [11–14]. Moreover, despite being expressed in multiple cell types in the brain, several loci have microglia-specific enhancers harboring AD-risk variants, such as the BIN1 loci [15, 16]. These findings have linked microglial activity to modification of AD risk. Thus, the exact mechanisms by which microglia contribute to AD continue to receive significant attention. In this review, we aim to summarize the genetic and functional discoveries that have advanced our understanding of microglia in AD. We will particularly focus on the role of triggering receptor expressed on myeloid cells 2 (TREM2) in amyloid, tau and demyelination pathologies.

## Microglia: from CNS development to homeostasis

**Fig. 1:**
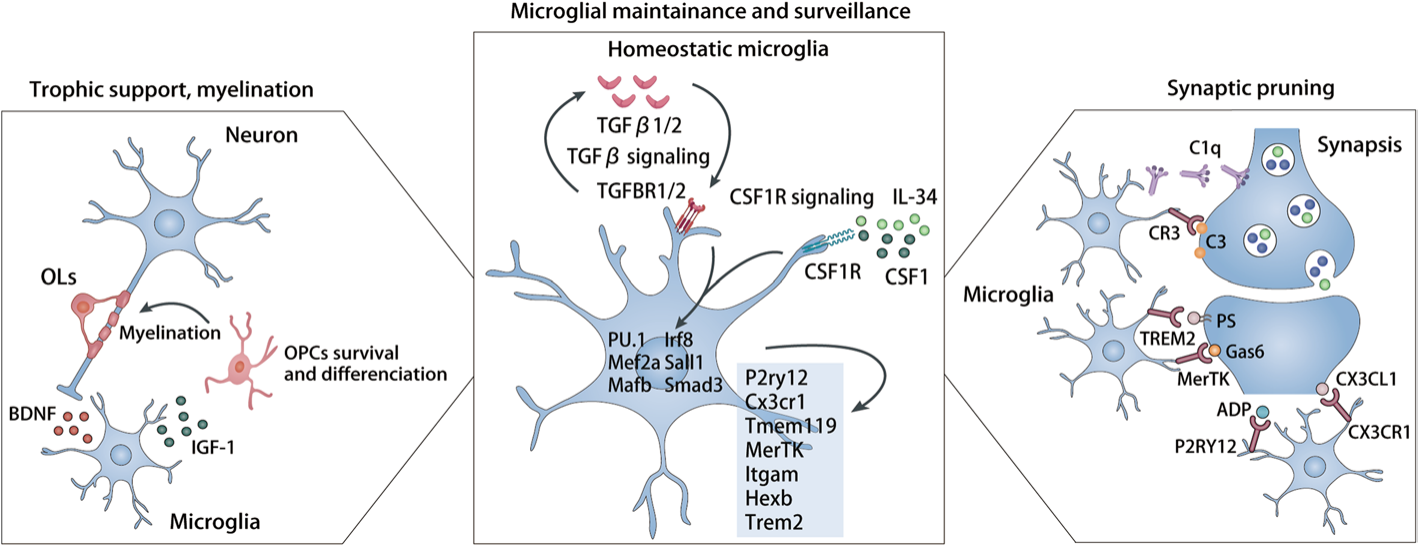
Microglial features in steady-state. In steady-state, microglia are maintained via paracrine colony-stimulating factor-1 receptor (CSF1R) signaling and via autocrine transforming growth factor-β (TGFβ) signaling. The two pathways endow a unique homeostatic feature of microglia, characterized by high expression levels of *P2ry12*, *Cx3cr1*, *Tmem119*, *MerTK, Itgam* and others. Microglia lineage-determining transcription factors PU.1, myocyte enhancer factor 2A (Mef2a), mothers against decapentaplegic homolog 3 (Smad3), transcription factors MafB (MafB), interferon regulatory factor 8 (Irf8), and spalt-like transcription factor 1 (Sall1) control microglia homeostasis. The features of homeostatic microglia make them well suited to the physiological functions, including neuronal trophic support via BDNF release, myelination via IGF-1 secretion, and synaptic pruning. Microglia remove excess synapses from postnatal or adult-born neuron via diverse ligand-receptor pathways including C3-CR3, PS-TREM2, Gas-MerTK, CX3CL1-CX3CR1, ADP-P2RY12, et al. OLs, oligodendrocytes; OPC, oligodendrocyte progenitor cell; IGF-1, insulin-like growth factor 1; BDNF, brain derived neurotrophic factor; CR3, complement receptor 3; TREM2, triggering receptor expressed on myeloid cells 2; MerTK, Mer tyrosine kinase; P2RY12, purinergic receptor P2Y; CX3CR1, C-X3-C motif chemokine receptor 1; C1q, complement component 1q; PS, phosphatidylserine; Gas6, growth arrest-specific 6; ADP, adenosine diphosphate; CX3CL1, C-X3-C motif chemokine ligand 1; TGFBR1/2, TGF-β receptor 1/2

Microglia arise from yolk-sac-derived precursors and maintain their population via local self-renewal in the healthy central nervous system (CNS) [17]. In mice, microglia emerge from early erythromyeloid precursor cells (EMPs) in the extraembryonic yolk sac at embryonic day 7.5 (E7.5), subsequently colonize the developing brain rudiment at E9.5 and go through three different phases, including early microglia (until E14), pre-microglia (from E14 to a few weeks after birth) and adult microglia (from a few weeks after birth onward) [18, 19]. These microglial phases show specialized gene expression profiles driven by the alterations in expression of lineage-determining and microglia-specific transcription factors [20–22], and require continuous instructive signaling from the brain, including transforming growth factor-β (TGF-β) signaling and colony-stimulating factor-1 receptor (CSF1R) signaling [23, 24] (Fig. 1). The early microglia phase is characterized by high expression of genes involved in cell cycle signaling pathways and colonization process and is driven by transcription factors such as E2f6, Klf2, Arid3a and Batf [18]; the pre-microglia phase is enriched for the expression of genes responsible for the neural maturation, synaptic pruning and axon tract development, and is driven by Rxrb and Fli1 [18, 25–31]. In this phase, the *Spp1*-expressing axon tract-associated microglia (ATM) and *Clec7a*-expressing proliferative-region-associated microglia (PAM) transiently appear in the developing white matter (WM) at post-natal day 4/5 and day 7 respectively and reflect the phagocytosis of apoptotic oligodendrocytes (OLs) [32, 33]. The adult microglia phase is maintained by transcription factors including Jun, Fos, Mef2a, MafB, MEF2a/c, Smad2/3/4, Irf8 and Sall1 [18], and is characterized by high expression of homeostatic markers such as the purinergic receptor P2Y12 (P2RY12), transmembrane protein 119 (TMEM119) and CX3C motif chemokine receptor 1 (CX3CR1), providing trophic support and modifying synaptic plasticity for neurons [34, 35] (Fig. 1). Most adult microglia are remarkably similar in transcriptomes and show limited regional heterogeneity. Studies of young adult mice found that the corpus callosum and the cerebellum contain a more immune-vigilant state of microglia than microglia in the cortex and striatum regions, which is correlated with an elevated degree of differentiating oligodendrocyte precursor cells (OPCs) apoptosis and neuronal attrition [36, 37]. These heterogeneous microglial populations may grant adult microglia the capacity of region-specific immunosurveillance but could also underlie region-specific vulnerability to microglial dysregulation in response to aging and disease [38, 39]. For example, cerebellar microglia are relatively more susceptible to aging compared to microglia of forebrain regions, exhibiting an earlier expression of type I interferon (IFN-I)-induced genes (*Irf7*, *Stat1*, *Osal1*, and *Bst2*) during aging compared to other regions [36].

In humans, microglia appear at gestational week 4.5 (GW 4.5) in the ventricular edge, the choroid plexus, and leptomeninges of the brain, then microglia colonize the telencephalon and diencephalon [40]. The transcriptomic profile of human fetal microglia during early and mid-gestation (GW9-18) development is similar to that of mouse microglia during development and exhibits a highly heterogeneous signature indicative of proliferative, activated, and phagocytic capacities [32, 33, 41], suggesting human microglia play similar roles in myelination and neuronal development [42, 43]. In adulthood, despite substantial overlap of microglial transcriptomic signature in humans and mice [44], adult human microglia show highly specific region-dependent gene expression profiles: microglia subset enriched in the subventricular zone and thalamus shows high expression of CD11c, CCR5, CD68, CX3CR1, HLA-DR, and the proliferation marker Ki67; microglia clusters enriched in temporal and frontal lobes express higher levels of CD206 [45]. The molecular machinery that drives human microglia development and regional diversity remain unclear. Also, whether these clusters of microglia in selected brain regions reflect a region-specific function or susceptibility of microglia to human aging and AD remains to be elucidated.

## Initial studies of microglia responses to AD pathology

During AD, the brain environment becomes highly perturbed locally, which leads to “reactive microgliosis”, characterized by microglia proliferation with a hypertrophic or ameboid shape [46]. Microgliosis proximal to amyloid plaques was initially identified from postmortem AD specimens and has long been considered a core component of neuropathology [47]. In accordance, mouse models of amyloidosis and tauopathy feature similar microglial expansion [48, 49]. Given the consensus that microglia are robustly activated during AD, extensive efforts have been devoted to understanding the breadth of microglial responses, and which of these responses are beneficial or detrimental in AD. Initial studies of microglial function in AD models have been focused on phagocytosis and release of pro-inflammatory mediators. Aβ has been postulated to directly activate CD36 signaling and other membrane receptors, such as toll like receptor (TLR) 2, TLR4, TLR6 and α6β1-integrin, which trigger phagocytosis and inflammatory response [50–56]. Additionally, the proinflammatory signature present in Aβ-plaque-associated microglia may partly be due to the activation of nucleotide-binding oligomerization domain-like receptor 3 (NLRP3) inflammasome. NLRP3 is a cytoplasmic sensor of cell injuries that forms the NLRP3 inflammasome together with procaspase-1 and apoptosis-associated speck-like protein containing a CARD (ASC) [57]. Early studies showed that CD36 could activate NLRP3 inflammasome by facilitating the internalization of Aβ [58]. The activation of NLRP3 inflammasome promotes proteolytic cleavage of dormant procaspase-1 into active caspase-1, and the release of mature IL-1β [59]. In an APP/PS1 model, *Nlrp3*^−/−^ and *Casp1*^−/−^ mice showed enhanced Aβ clearance and improved spatial memory [60]. Beyond a direct neuronal neurotoxic effect by IL-1β secretion, NLRP3 inflammasome also affects Aβ and tau pathology buildup. In vitro, assembled ASC specks could accelerate the Aβ aggregation through a cross-seeding activity [61]. In vivo, ASC specks co-sedimented with Aβ plaque and formed the core of plaques. Intrahippocampal injection of ASC specks exacerbated Aβ seeding in APP/PS1 mice [62]. Additionally, *Asc*^−/−^ and *Nlrp3*^−/−^ mice were resistant to tau hyperphosphorylation in the Tau22 model after injection of Aβ-containing brain homogenates [63], implying that the NLRP3 inflammasome is a potential therapeutic target for inhibiting both amyloid deposition and tau tangle formation during AD. However, evidence for NLRP3 relevance in human AD remains limited.

The pro-inflammatory function of microglia in AD progression has been corroborated by other studies. For example, ablation of interleukin-12 and interleukin-23 (IL-12/IL-23) results in attenuated Aβ pathology [64]. However, other studies showed that augmenting pro-inflammatory pathways by adeno-associated virus overexpression or intraperitoneal injection of tumor necrosis factor α (TNFα), interferon gamma (IFNγ), interleukin-33 (IL-33), interleukin-6 (IL-6), or by inhibiting interleukin-10 (IL-10), increase microglial phagocytotic activity resulting in reduced plaque deposition [65–69]. These seemingly opposing outcomes have suggested that microglia may have different impacts on AD pathology depending on the stage of disease. Consistent with this view, early microglia depletion by the CSF1R inhibitors PLX5622 or PLX3397 reduces plaque seeding in 4–5-month-old 5xFAD mice, a model of Aβ accumulation [70, 71], while microglia ablation does not alter the amyloid plaque load when treatment is initiated in 10-month-old 5xFAD mice after robust amyloid plaque deposition has already occurred [72].

## Heterogeneity of reactive microglia in AD

**Fig. 2:**
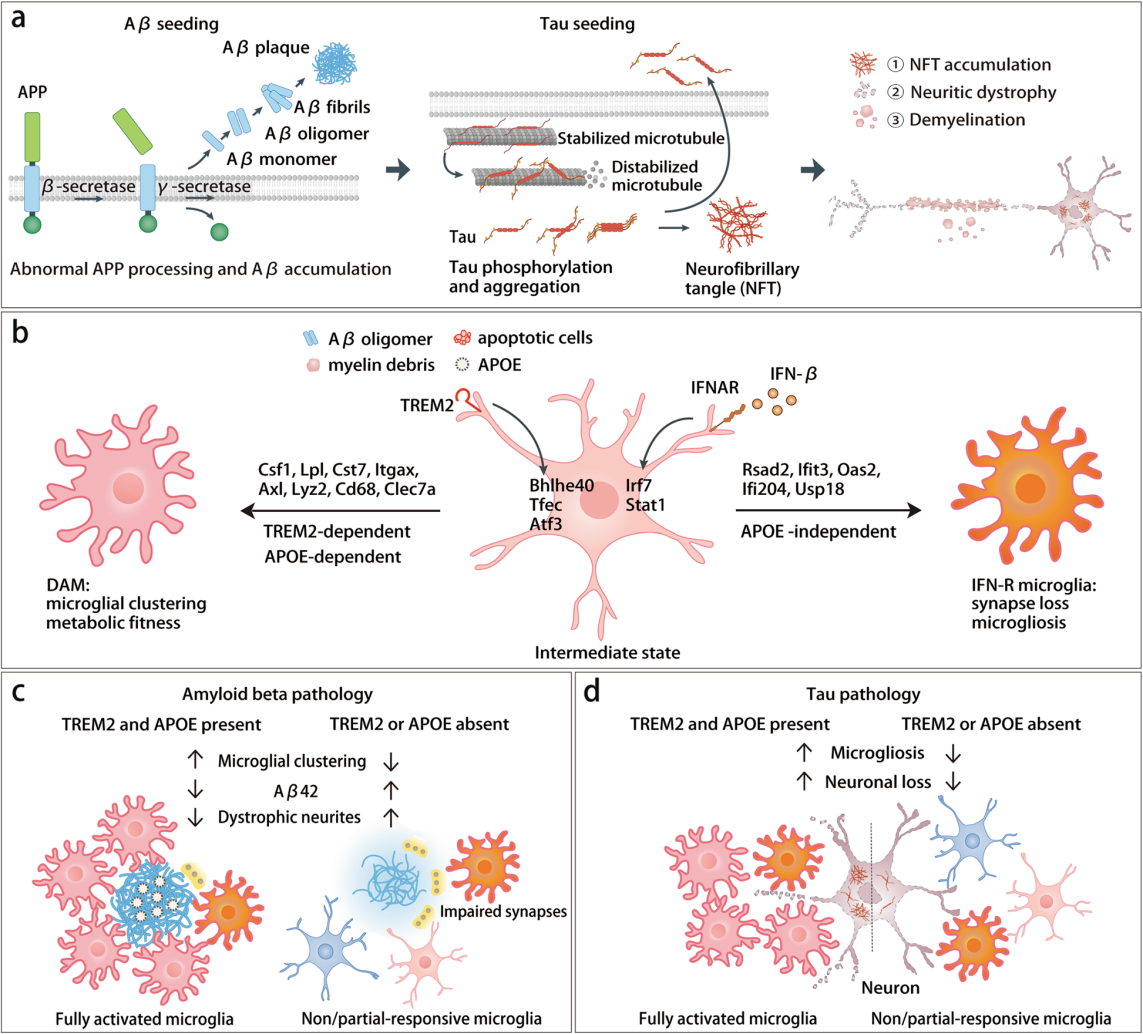
Microglial features in Alzheimer’s disease (AD). **a** AD is pathologically characterized by extracellular beta-amyloid (Aβ) aggregates, intracellular tau neurofibrillary tangles (NFT) and neuronal cell death. **b** In the setting of AD, microglia act as the initial responders to brain damage and respond to various pathological hallmarks, including Aβ oligomer, tau and myelin debris, adopting DAM and IFN-responsive microglia features via pathways that are in part TREM2 or APOE-dependent. Both DAM and IFN-R microglia are characterized by downregulation of homeostatic genes (such as *P2ry12*). DAM upregulate various genes, such as *Clec7a*. IFN-R microglia feature a list of up-regulated genes, including *Irf7* and *Stat1*. Transcriptional factors, such as *Bhlhe40*, *Tfec* and *Atf3*, are commonly induced in the reactive microglia through “TREM2-APOE” signaling. **c** In Aβ-bearing pathology, TREM2 and APOE collaboratively serve as a barrier to limit Aβ-induced neuronal toxicity by inducing microglial clustering. Lack of TREM2 or APOE results in a defect of DAM, and abundant Aβ42 and dystrophic neurites around filamentous plaques. **d** In tauopathy, TREM2 or APOE deficiency limits microgliosis and restrain neuronal loss in late-stage disease. DAM, disease-associated microglia; IFN-R, IFN-I-response

A major advance in understanding the “reactive microglia” has been achieved by high resolution transcriptome profiling. In early studies, the view of microglia activation was informed by the M1/M2 dichotomy originally proposed from macrophage polarization studies [73, 74]. It is now clear that the M1/M2 nomenclature does not embrace the complexity of microglia responses. Several bulk RNA-seq studies of microglia isolated either from spinal cord of the SOD1^G93A^ mouse model of amyotrophic lateral sclerosis (ALS), from the brain of 24-month-old mice, from cuprizone-induced demyelination, and from the Aβ-overexpressing 5xFAD models confirmed that the microglial activation signature is much more complex than the simple M1/M2 dichotomy [75–78]. With the help of the recently developed single cell (scRNA-seq) technology, the transcriptional profile of microglia in the Aβ-bearing 5xFAD mouse model was clearly defined [79]. This approach led to the identification of disease-associated microglia (DAM), which are characterized by downregulation of homeostatic genes (*Hexb*, *P2ry12*, *Tmem119* and *Tgfbr1*), and upregulation of genes involved in inflammation (*Il-1β*, *Ccl6*), phagocytosis (*Trem2*, *Tyrobp*, *Axl*), cell survival (*Csf1*, *Igf1*), lysosome function (*Cst7*, *Cd68*, *Cstb/d*, *Lyz2*) and lipid metabolism (*Apoe*, *Lpl*, *Ch25h*). This signature was confirmed by bulk RNA-seq of microglia in models of AD (APP/PS1 mice), ALS, and multiple sclerosis (MS) and referred to as a “microglial neurodegenerative” (MGnD) phenotype [80]. Transition of homeostatic microglia to DAM is partially dependent on the microglia receptor TREM2 (Fig. 2) [79]. Beyond the DAM, additional reactive microglial populations have been identified in models of neurodegeneration. scRNA-seq of microglia in the inducible CK-p25 mouse model of neurodegeneration has revealed two additional reactive microglia phenotypes in late response to neurodegeneration, typified by high expression of IFN-I-response (IFN-R) genes, such as *Irf7* and *Oas1a*, and MHC class II (MHC II) genes such as *Cd74* and *H2-Aa* [81]. A fourth microglial signature expressing markers of proliferation (Cycling microglia, Cyc-M) was further identified [82, 83]. All microglia signatures (DAM, IFN-R, MHC II and Cycling) were subsequently confirmed by meta-analysis in mouse models of Aβ accumulation, tauopathy and demyelination, where the signatures either coexist or are overshadowed by the DAM signature [84]. Trajectory analysis addressed the relationships among all microglial subsets in 5xFAD model, showing that microglia progressively transition from a homeostatic state into four distinct populations in TREM2-dependent manner: DAM, IFN-R, MHCII, and Cyc-M (Fig. 2) [83].

Similar microglial sub-populations have been corroborated in the APPNL-G-F knock-in (KI) model with different nomenclatures: activated response microglia (ARMs), transiting response microglia (TRMs), interferon response microglia (IRMs) and cycling/proliferating microglia (CPMs) [85]. ARMs were enriched for expression of tissue repair genes (*Gpnmb*, *Spp1* and *Dkk2*), which were also found in DAM. In this APPNL-G-F KI model, homeostatic microglia progressed toward the ARM state in an APOE-dependent manner, whereas conversion to IRMs was APOE-independent (Fig. 2) [85]. Beyond AD models, DAM-like signatures have been found in other contexts. The PAM subset transiently appearing during the development of mouse brain WM, shares a characteristic gene signature with DAM/ARM, although PAM do not depend on the TREM2 or APOE [33]. Safaiyan et al. observed a “white-matter associated microglia (WAMs)” signature in aging mice that is TREM2-dependent [86]. Qiu et al*.* observed that a genetic defect of cerebroside sulfotransferase, which causes a mild adult-onset loss of myelin sulfatides without disrupting myelin homeostasis, is sufficient to induce DAM-like signature [87]. Overall, these signatures may reflect the occurrence of myelin alteration, remodeling, or degeneration, which stimulate the differentiation of DAM-like microglia capable of maintaining myelin integrity.

To specifically focus on Aβ-induced microglia signatures, Grubman et al*.* and Xia et al*.* isolated two different populations of microglia based on their content of fibrillar Aβ (fAβ) labeled with methoxy-XO4 (methoxy-XO4-positive and methoxy-XO4-negative microglia) from the 5xFAD model [88] and the App^SAA^ KI/KI model [89], respectively. Analysis of the transcriptomes of the XO4-positive microglia revealed a hypoxia-inducible factor 1α (HIF-1α)-driven transcriptional signature, which partly overlapped with the DAM [88, 89], and displayed a higher phagocytic ability in vitro [88]. Lipidomic and metabolomic analyses showed a prominent accumulation of neutral lipids and a selective accumulation of the polyamine spermine in the methoxy-XO4-positive microglia [89]. Moreover, Xia et al*.* confirmed that most methoxy-XO4-positive microglia were found around amyloid, indicating the microglial response depends on the proximity to the amyloid plaques [89]. In support of this, the gene expression signature associated with XO4-positive microglia did not persist after 5xFAD microglia were exposed to wild-type (WT) organotypic hippocampal slice cultures for 5 days [88], suggesting that either the XO4-positive transcriptional program reverted upon digestion of internalized fAβ, or the original XO4-positive microglia died. This observation will prompt further studies on the fate of DAM using the fate-mapping tools. By contrast, XO4-negative microglia seemed to be more neurotoxic because they contained more intracellular postsynaptic material than XO4-positive microglia and appeared to age faster than do microglia in age-matched normal brain [88]. XO4-negative microglia displayed reduced phagocytosis ex vivo [88]. Because aged microglia have reduced phagocytic activity and are prone to sustain disease progression, they could represent an interesting target for AD therapeutics.

Rexach et al*.* concentrated their analyses of microglia responses in different stages of tauopathy models by bulk tissue RNAseq, and found two modules that encompass opposing signaling pathways occurring in early responses: the neuroimmune activation (NAct) module expresses activation markers, such as complement, NLRP3 inflammasome, TLRs, NF-κB targets; by contrast, the neuroimmune suppression (NSupp) module expresses markers of immune suppression and exposure to type I IFN [90]. Based on these data, it has been proposed that TLRs and NOD-like receptors elicit the NAct module in an initial stage of early response to tauopathy; as disease progresses causing the release of nucleic acids, cytosolic detection of nucleic acids elicits the NSupp module and type I IFN signaling, which in turn partially suppresses the NAct module. This sequence mimics that occurring in pathogenic infections in which initial immune activation is followed by a type I IFN response with immunoregulatory functions [91]. The NAct and NSupp modules were found to be conserved in human samples of Pick tauopathy analyzed by snRNAseq. Finally, Rexach et al*.* showed that beyond the bimodular early phase, an IFNγ signature was detectable at late stages of disease. Overall, this study suggest that phenotypic responses of microglia vary depending on the type and duration of pathological events and that disease progression may be caused by early immune suppression, rather than immune activation alone. A finer characterization of microglial signatures in mice carrying various AD pathologies at different stages and carrying different LOAD risk variants may be important for choosing microglia-based therapeutic strategies and intervention windows.

The discovery of transcriptionally heterogeneous populations of microglia responding to AD pathology raises the questions of how these subsets are generated and maintained and what is their impact in the progression of the disease. It has been shown that DAM secrete CSF1, which may elicit an autocrine-paracrine feedback loop through CSF1R, sustaining proliferation and survival of microglia around plaques [92, 93]. By contrast, type I IFN acts as an endogenous inhibitor of CSF1-stimulated macrophages and attenuates cell cycling [94]. The IFN-R subpopulation is associated with an increase in neuroinflammation and cognitive decline during aging and AD [32, 95–98]. Accordingly, blockade or ablation of the receptor for type I IFN (IFNAR) abolished the upregulation of IFN-responsive genes in aging or AD mouse models [96, 98, 99], diminished microgliosis and synapse loss in AD models [95], and partially reduced age-related cognitive decline [99]. Therefore, different microglial subsets and their underpinning molecular circuitries may have either beneficial or detrimental impacts on AD brain, and the overall impact of microglia on disease progression depends on the net effect of those subsets.

## Discrepancies between human and mouse microglial signatures in AD

Human microglial signatures obtained from scRNA-seq/single nucleus RNA-sequencing (snRNA-seq) of AD brain specimens demonstrate considerable variability and seem to be in part inconsistent with murine DAM signatures, as discussed in a recent review [100]. These inconsistencies maybe caused by the heterogeneity of age, gender, and disease stage among study subjects and by the methodology used in sample processing [101, 102]. To address the non-negligible transcriptional differences that exist between the humans and mice, Hasselmann et al. and Mancuso et al*.* developed a chimeric model to study and manipulate human microglia in humanized mouse models [103, 104]. First, they transplanted either the human induced pluripotent stem cells (iPSCs)-derived hematopoietic-progenitors or human embryonic stem cells (ESCs)-derived microglia into the postnatal brain of humanized, immune-deficient mice (MITRG model or human M-CSF KI model). They confirmed that xenotransplanted microglia (xMGs) closely resemble in vivo homeostatic human microglia signature, expressing key microglia-associated transcription factors (e.g., SALL1) and homeostatic genes (e.g., P2RY12, CX3CR1 and TMEM119) [103, 104]. Hasselmann et al*.* further characterized the xMGs response to amyloid pathology at the transcriptional level by isolating xMGs from the brains of aged MITRG mice and 5xFAD-MITRG mice and observed that plaque-associated xMGs upregulate markers including APOE, CD11c and TREM2, which are consistent with the murine DAM signature [103]. TREM2-deficient xMGs or xMGs carrying the TREM2-R47H variant associated with high AD risk showed defects in clustering around amyloid plaques, phagocytosis, and expression of DAM markers [105, 106]. By contrast, Mancuso et al*.* observed that human ESCs-derived microglia and endogenous mouse microglia from the same animal displayed a divergent transcriptomic response in response to acute intracerebroventricular administration of oligomeric Aβ: the mouse microglial cluster showed a profile similar to murine DAM [104]; while the human microglial cluster displayed high levels of multiple inflammatory cytokines and chemokines, such as IL-1β, IL-6, CCL2 and CCL4. Collectively, these studies indicate that the pathways underlying acute and chronic responses of human and mouse microglia to oligomeric Aβ and Aβ plaques may be different. The efficient engraftment and integration of human iPSCs/ESCs-derived microglia in vivo in humanized mice models will greatly help to determine how human microglia respond to progressive AD pathology, to identify signaling and transcriptional pathways that guide microglia phenotypic diversity, and to determine the effect of risk variants defined by GWAS in AD pathology.

## Microglia response to myelin damage during AD

In addition to extracellular Aβ plaques and intracellular NFT, Alzheimer originally reported yet another feature of AD pathology, which was defined as an extensive accumulation of “adipose saccules” in glial cells [47]. The “adipose saccules” likely correspond to what we now term lipid droplets (LDs), which are usually composed of neutral lipids, such as cholesteryl esters (CE) and glycerolipids [107]. The “glial cells” are likely dysfunctional microglia containing LDs. This feature may reflect microglia phagocytosis of myelin debris and lipids that are released due to myelin damage, especially in the WM. Indeed, AD has been shown to associate with loss of myelin integrity and alteration of lipid content [108]. Human neuroimaging studies have detected changes in the volume of WM hyperintensities early in AD, which are predictive of disease status [109–111]. Pathological studies observed a striking decline of myelin density in WM and an association of Aβ deposition with focal intracortical demyelination in gray matter [112–114]. Additionally, gene products related to myelin and oligodendrocyte modules, such as PICALM, were enriched within AD risk loci [115, 116]. snRNA-seq of 5xFAD brains showed an unusual *Serpina3n* + *C4b* + reactive oligodendrocyte population, which may indicate an oligodendrocyte response to myelin dysfunction during Aβ deposition [117]. This observation has been further supported by a spatial transcriptomic study that found expression of oligodendrocyte response gene around Aβ plaques in App^NL−G−F^ mice [118].

In addition to AD, LDs-bearing microglia have also been observed in models of demyelination, such as that induced by the demyelinating agent cuprizone (CPZ) [119], as well as in brains of aging mouse and human, and termed as lipid-droplet-accumulating microglia (LDAM) [120]. LDAM exhibit phagocytotic dysfunction, increased production of reactive oxygen species and proinflammatory cytokines [120]. Moreover, LDAM transcriptional signature is distinct from that of DAM in AD: some of genes upregulated in DAM were downregulated in LDAM (for example, *Axl*, *Cd74* and *Clec7a*) [120]. LDs found in LDAM contain predominantly neutral lipids such as glycerolipids and little CE.

Depp et al*.* found that defective myelin is an upstream factor of Aβ deposition [121]. They examined Aβ-bearing mice with myelin defects ranging from myelin deterioration (*Cnp*^−/−^, *Plp*^−/y^, CPZ model, EAE model) to depletion of compact myelin (*Emx-Cre* x *Mbp*^flox/flox^) and observed that Aβ deposition could either be induced or reduced, respectively [121]. *Cnp*^−/−^ x 5xFAD mice in particular lacked plaque-clustering microglia, suggesting that microglia, once engaged in the clearance of myelin debris, are distracted from Aβ clearance [121]. Thus, these results suggest that microglia prioritize ingesting myelin debris-lipids versus Aβ in the AD brain. Moreover, variants of proteins involved in lipid sensing and cholesterol transport, such as TREM2 and APOE, may increase LOAD risk by impacting lipid metabolism [4, 122, 123].

## Rare TREM2 variants increase the risk of neurodegeneration

TREM2 is a single-pass transmembrane receptor which belongs to the Immunoglobulin superfamily (Ig-SF). It is predicted to consist of an extracellular V-type Ig domain (IgV), a long stalk, a single transmembrane helix that associates with the adaptor protein DAP12 via lysine-aspartic acid interaction, and a short cytosolic tail that lacks any signal transduction motif [124]. DAP12 binding is required for both TREM2 membrane stabilization and downstream signaling [125, 126]. Natural ligands of TREM2 include anionic and zwitterionic phospholipids and glycolipids, apoptotic cells exposing phospholipids, lipidated particles (e.g., HDL and LDL), and lipoproteins (e.g., APOE and CLU/APOJ) [127]. TREM2 can also bind Aβ oligomers [128–131]. TREM2 is expressed in tissue macrophages including microglia within the brain. One characteristic of TREM2 is the shedding of the ectodomain through cleavage mediated by ADAM10/17 at histidine 157 (H157) within the stalk region, which results in the generation of soluble TREM2 (sTREM2) [132–134]. Rare variants of TREM2 that cause complete lack of TREM2 expression were first reported in Nasu-Hakola disease (NHD), an autosomal recessive early-onset dementia with bone cysts [135]. Almost a decade later, two parallel GWAS identified heterozygous rare variants of TREM2 that are associated with higher risk of developing LOAD [122, 123]. The most common TREM2 variant, *R47H*, was shown to increase the risk of AD by twofold to threefold, and additional TREM2 variants, such as *R62H*, *T66M*, *H157Y*, *D87N*, also increase the risk of LOAD by affecting TREM2 expression, surface trafficking, shedding, ligand binding or signaling [136–140]. The genetic association of TREM2 variants with AD has prompted in depth studies on the impact of TREM2 on microglia responses to pathological features of AD, including Aβ plaques, tauopathy and demyelination, which will be reviewed in the following sections. The broad role of TREM2 on macrophage functions in neurodegeneration, obesity, and cancer [77, 141–143] has been addressed in a recent review [144].

## TREM2 sustains microglial response to Aβ

TREM2 is constitutively expressed in microglia, but its expression ramps up after initiation of amyloid plaques [145]. Initial studies in the 5xFAD model of Aβ plaques revealed that TREM2-deficiency impaired microglial clustering and activation around Aβ plaques, increasing Aβ load and reducing the density of Aβ plaques [77]. Plaques were more diffuse, had larger surface and appeared more filamentous than compact [77, 146, 147], which resulted in a greater dystrophy of axons and dendrites (Fig. 2) [148]. These results suggested that TREM2 was necessary for the generation of plaque-associated microglia, which form a barrier that limits spreading and neuronal toxicity Aβ. Indeed, patients carrying *TREM2 R47H* variant and transgenic mice expressing the same variant in place of endogenous *Trem2* revealed similar patterns of microglial clustering defects and plaque morphology [149, 150]. Further supporting these findings, increasing TREM2 gene dosage by overexpression of human TREM2 in 5xFAD mice expressing endogenous *Trem2* (5xFAD/BAC-TREM2) alleviated Aβ deposition [151]. Reduction of plaque load was associated with decreased activation of microglia which exhibited enhanced ramified morphology [151]. Moreover, systemic administration of high doses of agonistic antibodies against TREM2 reduced Aβ load, increased plaque compaction, reduced neurite dystrophy, and improved behavioral performance [82, 83, 152]. Altogether, these studies support the potential therapeutic benefit of activating TREM2 in AD. Profiling of microglia transcriptome in mouse models of Aβ plaques accumulation lacking TREM2 or expressing the R47H TREM2 variant showed that microglia require TREM2 to acquire the typical DAM signature (Fig. 2) [79, 82]. The inability of *Trem2*-deficient or loss-of-function (LOF) mutation microglia to cluster around plaque and acquire a DAM signature is in part due to impaired activation of the mTOR pathway, which results in reduced protein synthesis and energy metabolism [153–156]. These findings indicate that microglial activation and metabolic fitness are critical components that sustain microglia defense against Aβ pathology.

Some studies have proposed that the impact of TREM2-mediated microglia activation varies with the disease stage. One study found that TREM2 deficiency was associated with reduced Aβ load at early stage of plaque formation [145], whereas Aβ load was increased late in disease [147]. Another study generated a TREM2 overexpression mouse through expression of a TREM2 transgene resistant to shedding by ADAM10/17 protease by mutating the protease recognition site in the stalk region [157]. Overexpression of non-cleavable TREM2 in APP23/PS45 mouse model (APP23/PS45/IPD) resulted in increased numbers of small plaques but not medium and large plaques in the cortex compared to control mice by 3-month-old of age. Of note, APP23/PS45/IPD mice showed lower levels of soluble Aβ and higher levels of insoluble Aβ in forebrain compared to APP23/PS45 mice. These observations are in line with the reported role of other microglial receptors, such as MERTK and AXL, in facilitating dense-core plaque formation [158] and the effect of TREM2 agonistic antibody in decreasing the number of filamentous neurotoxic plaque [82]. The APP23/PS45/IPD mice also exhibited increased expression of cytokines and chemokines in the forebrain lysates, suggesting that TREM2 overexpression promotes secretion of inflammatory factors that consequently might facilitate plaque seeding in the initial stage of Aβ accumulation [145, 157].

In contrast to all the above studies, a recent report showed that short-term administration of *Trem2* knockdown antisense oligonucleotides (ASOs) through the ventricles in 10-month-old APP/PS1 mice that exhibit widespread plaques and highly activated microglia, reduced plaque load by half. Conversely, injection *Trem2* knockdown ASOs at pre-plaque (4-month-old) and early-plaque stages (7-month-old) did not affect plaque load [159]. Given the potential immunoreactivity and limited brain biodistribution of ASOs, further studies are required to support this concept. Overall, these studies indicate that the impact of TREM2 on Aβ pathology may vary at different disease stages and therefore the efficacy of TREM2-based therapeutics may be restricted to a certain time window.

## TREM2 in tauopathy

Opposing roles for TREM2 have been reported in mouse models of pure tauopathies. Leyns et al*.* found PS19/*Trem2*^−/−^ mice display substantially less atrophy than PS19/WT mice at 9-month-old, as judged by little ventricular enlargement and less synaptic loss, although the amount of phosphorylated and aggregated tau did not differ [160]. The PS19/*Trem2*^*−/−*^ mice showed much less microgliosis and astrogliosis in the hippocampus and piriform cortex than PS19/WT controls, and their microglia expressed fewer markers of DAMs and inflammation. Similarly, Sayed et al*.* observed that TREM2 deficiency prevented microglial activation and hippocampal atrophy in 9-month-old PS19 mouse, while *Trem2*^+/-^ mice showed exacerbated tau pathology [161]. Gratuze et al*.* crossed the PS19 mice lacking endogenous mouse TREM2 with mice expressing the common variant (CV) of human TREM2 (TREM2 CV) or the TREM2 R47H variant [162]. By 9 months, PS19/TREM2 R47H mice showed significantly alleviated tauopathy versus PS19/TREM2 CV mice. Microglia were more quiescent in the R47H mice versus CV mice, as evidenced by lower expression of DAM genes, the phagolysosome marker CD68 and complement component C1q. These observations paradoxically suggest that the TREM2 LOF mutation protects against neuronal loss by tuning down microglial activity in tauopathy. By contrast, Bemiller et al*.* showed that TREM2 has a protective role in disease by restraining tau hyperphosphorylation and aggregation in a weaker tauopathy model [163]. Bemiller et al*.* crossed *Trem2*^−/−^ mice with the hTau^+/-^/mTau^−/−^ mouse model that carries normal human Tau under control of the human microtubule associated protein tau (MAPT) promoter in place of the murine endogenous gene. In this model, Tau becomes hyperphosphorylated at around three months of age and starts to aggregate at around 6 months. By 6 months of age, results showed that TREM2 deficiency enhanced tau phosphorylation and stress signaling in the brain. In line with this study, TREM2 ablation aggravated synaptic loss also in a model of frontotemporal lobar degeneration [164].

Recent work further examined how TREM2 impacts pathology when Aβ and tau coexist. Encapsulation of plaques by microglia was inversely correlated with amyloid-β42 (Aβ42) ‘hot-spots’ on plaques [165]. Aβ42 is neurotoxic and can induce tau phosphorylation and microtubule instability [166]. TREM2 deficiency, the TREM2 R47H variant, or microglia depletion caused not only a significant increase of Aβ42 around plaques in APPPS1-21 mice but also increased seeding and spreading of human tau derived from AD patients that was directly injected into the APPPS1-21 brain [167, 168]. Similarly, Lee et al*.* examined *Trem*2-deletion in the pR5-183 mouse model expressing P301L mutant tau alone (Tau P301L) or in combination with the PS2/APP transgene (Tau/PS2/APP), and found that *Trem*2-deficiency exacerbates tau propagation and consequent brain atrophy in the Tau/PS2/APP model, but not in the Tau P301L model at both early and late degenerative stages [169]. scRNA-seq showed a blunted DAM activation by *Trem*2-deficiency in Tau/PS2/APP model compared to WT Tau/PS2/APP mice, indicating TREM2-dependent acquisition of DAM limits tau propagation in Tau/PS2/APP model [169]. Interestingly, *Trem2*^+/+^, *Trem2*^+/-^, *Trem2*^−/−^ mice showed comparable changes in tau pathology in a 17-month-old pure tauopathy model [169]. This finding corroborates that TREM2 may suppress tau seeding in early phases but aggravate tau propagation later in AD [160–163].

## TREM2 controls microglia processing of myelin and lipid droplets

Microglia have a prominent role in CNS myelination during development, homeostasis, and pathologies that cause myelin injuries: they clear myelin debris, allowing replacement of damaged myelin with newly formed myelin, and reducing the load of oxidized lipids [119, 170]. As lipid sensor, TREM2 detect myelin components, such as sulfatides, phospholipids, or oxidized phospholipids, and deliver intracellular signals that sustain phagocytosis and induce microglia metabolic programs, including lipid catabolism and cholesterol efflux. Several studies in models of demyelination corroborate a requirement of TREM2 for these microglial functions. Mice with *Trem2* deficiency treated with CPZ showed defective clearance of myelin debris and remyelination [78, 171]. In this model, TREM2 deletion impaired the expression of lipid catabolism genes [78, 171], provoked microglial accumulation of LDs and biased LDs composition towards the accumulation of CE and their oxidized derivatives [119]. Likewise, iPSC-derived microglia-like (iMG) cells expressing TREM2 R47H also showed accumulation of CE in response to incubation with myelin debris in vitro [172]. Corroborating TREM2 function in facilitating cholesterol disposal, deletion of other molecules along the TREM2 pathway, such as APOE and phospholipase Cγ2 (PLCγ2), also resulted in the buildup of CE in microglia or iMG upon incubation with myelin debris [119, 172, 173]. Although CE accumulation has been observed in the brains of AD patient and AD mouse models [174], it remains unclear how much CE and oxidized CE species derived from abnormal accumulation of CE are neurotoxic and contribute to AD pathology.

In apparent contrast with these studies, there is evidence that TREM2 may promote cholesterol esterification and generation of LDs in response to myelin debris uptake, which may be beneficial in buffering cellular free cholesterol-mediated toxicity [175]. Gouna et al*.* found that TREM2-deficient microglia were unable to generate LD-containing microglia in the lysolecithin (LLC)-induced model of demyelination, resulting in reduced LDs biogenesis and buildup of endoplasmic reticulum stress 21 days post LLC injection (dpi) [175]. In this model, locally injected LLC creates an acute demyelinated lesion within 4 dpi [176]. In contrast, oral administration of CPZ creates massive demyelination in the corpus callosum after 4–5 weeks of CPZ feeding [177]. Hence, LLC-induced demyelination is more aggressive than that induced by CPZ, such that TREM2-deficient microglia may be unable to adapt to fast myelin debris exposure. A defect of LDs biogenesis in TREM2 LOF microglia was also observed in Aβ pathology. Claes et al*.* transplanted TREM2 CV and TREM2 R47H iPSC-derived microglial progenitors into the 5xFAD–hCSF1 mouse model. By 7-month-old, the TREM2 R47H xenografted microglia (xMGs) exhibited reduced reactivity to plaque and reduced accumulation of LDs in vivo, without affecting the Aβ load [106]. Overall, these conflicting studies will prompt further dissection of the role of TREM2 in myelin clearance and lipid metabolism as well as the possibility to harness TREM2 in the treatment of demyelinating diseases. An encouraging observation in this direction is that the administration of an agonistic TREM2 antibody promoted myelin clearance and remyelination in a model of multiple sclerosis [178].

## TREM2-APOE interactions

The link between APOE and TREM2 has been extensively investigated. Homeostatic microglia produce low levels of APOE in the steady state. However, DAM produce high levels of APOE in human AD and mouse AD and this production is partly TREM2 dependent [79, 80, 117, 179]. Microglia secreted APOE is an important constituent of amyloid plaques that promotes their compaction. Accordingly, plaque-associated APOE was markedly reduced in *Trem2*^−/−^ mice, TREM2 T66M mice, or in a genetic model of microglial ablation [139]. Moreover, a direct interaction between TREM2 and APOE has been reported in vitro by biochemical approaches [180, 181]. Thus, plaque-associated APOE produced by microglia may in turn facilitate TREM2 activation by Aβ plaques [182–184]. Indeed, amyloid plaques in both APOE- and TREM2-deficient mice show similar microglia clustering defect, lack of compaction, filamentous morphology and association with neurite dystrophy [184] (Fig. 2). Moreover, acquisition of the DAM signature is in part APOE-dependent [85]. These findings support that TREM2-APOE interaction may contribute to induce the switch from homeostatic microglia to DAM in AD [139]. Interestingly, McQuade et al. observed that APOE variants are recognized and engulfed by TREM2 at different rates with APOE4 > APOE3 > APOE2 [105], suggesting that APOE4 may have a major impact on microglia than other APOE isoforms because of a stronger interaction with TREM2.

The TREM2-APOE axis has also been investigated in tauopathy and demyelination. Mice lacking TREM2 [160, 162] or APOE [185–187] exhibit similar reduction in tau pathology in P301S model at advanced disease stages (9-month-old), although the mechanisms underlying these similarities are not fully elucidated (Fig. 2). In demyelination models, *ApoE* upregulation in microglia was TREM2-dependent [119]. Loss of *Apoe* caused intracellular accumulation of CE in microglia that exhibited a foamy phenotype [119, 173], which was also observed in *Trem2*-deficient microglia and in human iMG lacking either TREM2 or its signalling downstream effector PLCγ2 [119, 172]. Overall, these findings establish reciprocal links between APOE and TREM2 in many microglial functions in AD and demyelinating disorders.

## Structural insights into the human TREM2 variants

**Fig. 3:**
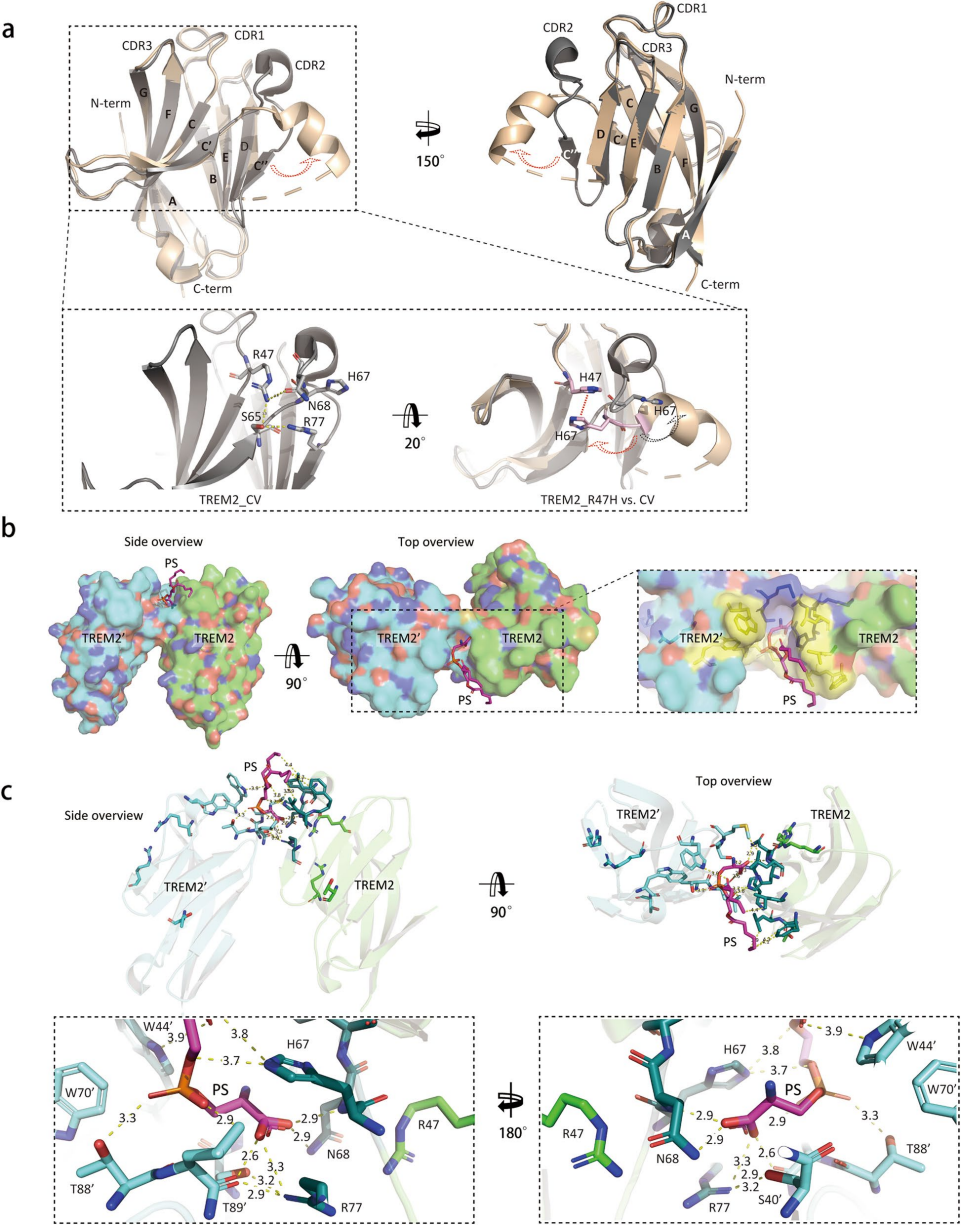
Structural insights into TREM2 ligand recognition and the impact of LOF mutation. **a** Structural alignment of TREM2 CV (PDB: 5UD7) vs. TREM2 R47H (PDB: 5UD8). The top cartoon depiction shows the overall structure of TREM2 R47H (in beige) superposed with TREM2 CV (in grey). The β-strands and CDR loops of Ig-like domain of TREM2 were labeled from A to G and from CDR1 to CDR3, respectively. The red dashed arrows indicate the conformational change of CDR2 loop caused by R47H mutation. At the bottom, the left panel of the zoom-in image shows that R47 is involved in an interacting network with S65, N68 and R77 to stabilize regular TREM2 Ig folding of the C’’ strain and CDR2 loop in TREM2 CV. The right panel of the zoom-in image illustrates that H47 (in pink) forms a new aromatic π–π stacking with H67 (red dashed line), leading to a turning of H67 (red dashed arrow) from normal orientation (grey H67) and CDR2 conformation change (black dashed arrow). **b** The embedded phosphatidylserine (PS) binding pocket is created by two adjacent TREM2 molecules (TREM2 and TREM2’). The original structures of TREM2 are shown with electrostatic surface area and PS molecule is presented with sticks for better visualization (modified from PDB: 6B8O). Green indicates the surface of TREM2, and Cyan indicates the surface of TREM2’. Blue highlights indicate the NH groups and red highlights the CO groups of the protein. In the zoom-in image, blue sticks/surface show the hydrophilic residues interacting with PS head groups, and yellow sticks/surface illustrate the hydrophobic neck of the ligand binding pocket. **c** The structural model of PS binding to TREM2 dimer (modified from PDB: 6B8O). Highlighted sticks in the zoom-out panel show all the putative interactions and the physical distances between interacting atoms (dashed bar) in the PS binding sites. Dark green sticks show the residues from TREM2 interacting with the PS molecule; light cyan sticks show the residues from TREM2’ interacting with the PS molecule. Residues with apostrophe marks are from the second TREM2 molecule (TREM2’)

The structural basis for the LOF TREM2 variants has been extensively studied. The classic IgV-like folding of TREM2 ectodomain was shown to form a gulf that contains many positively charged residues, including two sites affected by LOF variants, R47H and R62H, which was predicted as the ligand binding pocket [124]. The putative ligand binding sites were confirmed later on by the structure from Sudom et al*.* [188]. In this study, the TREM2 R47H structure revealed that the R47H mutation introduces a novel configuration with an aromatic π–π stacking between H47 and H67 thatdisrupts C’’ and complementarity-determining region (CDR) 2 conformations of TREM2 CV, which can potentially interfere with binding of phosphatidylserine (PS) (Fig. 3a). A caveat is that the TREM2 R47H protein in Sudom et al. structure was expressed in an *Escherichia coli* system that precludes glycosylation [188]. TREM2 is heavily glycosylated in mammalian cells and in vivo [124]. One of the two glycosylation sites on TREM2 is at N79 site which locates exactly at C’’ end. Therefore, whether secondary structure changes were due to the loss of the glycosylation remains to be determined. However, if the TREM2 R47H structure is correct, it may provide a mechanism explaining the LOF caused by R47H mutation. In accordance with such model, a reporter cell assay showed that TREM2 R47H activation by PS was strongly attenuated but not completely lost [77, 150].

Sudom et al*.* also resolved the structure of TREM2 CV with a bound PS ligand, which unveiled a surprising ligand binding pocket that is different from the predicted pocket where R47 and R62 are located [188]. In this structure, TREM2 forms a tight dimer that generates the deep binding pocket for the insertion of one PS molecule (Fig. 3b): the hydrophilic head of PS is engulfed by surrounding hydrophilic residues (highlighted in blue); the hydrophobic tail is further stabilized by the hydrophobic surface outside the pocket (highlighted in yellow) (Fig. 3b). The molecular interactions with PS are mainly contributed by H67 and N68, along with L72, F74 and L75 (not shown) from one TREM2 and the W44’, T88’, and L89’ from another TREM2’ (Fig. 3c). This observation further supports the theory that TREM2 signaling requires clustering of the TREM2-DAP12 complex on the cell surface.

## Soluble TREM2

One feature of TREM2 is the shedding of the ectodomain by ADAM10/17-mediated cleavage of TREM2 at H157, which results in the generation of sTREM2 [132–134, 189]. A TREM2 H157Y variant that is associated with increased risk of LOAD in the Han Chinese cohort leads to enhanced shedding of TREM2 [138], implying that reduced surface expression of TREM2 and/or increased sTREM2 may augment AD risk. However, 5xFAD mice carrying the TREM2 H157Y exhibited reduced amyloid pathology, suggesting TREM2 H157Y might impact AD risk through an amyloid-independent pathway, such as its effects on tauopathy [190]. Consistent with this conclusion, variants of tetraspanin MS4A associated with reduced AD risk were shown to correlate with increase sTREM2 in the cerebrospinal fluid (CSF) suggesting a protective role of sTREM2 [191, 192]. Thus, it remains unclear whether more TREM2 cleavage is beneficial or detrimental in AD. TREM2 cleavage and sTREM2 may impact multiple processes. First, TREM2 cleavage prevents TREM2 ability to transduce intracellular signals, also because sTREM2 may function as a decoy receptor for ligands that trigger signaling of full length TREM2. Second, sTREM2 can impact Aβ pathology: sTREM2 binds amyloid aggregates [150] and can contribute to compacting amyloid plaques, reducing diffuse neurotoxic Aβ plaques that damage adjacent neurons [146]. Third, some reports have indicated that sTREM2 can trigger an independent signaling pathway, presumably by activating extracellular signal-regulated kinase 1/2 (ERK1/2) pathway [193–195]. Accordingly, exposure of bone marrow-derived macrophages and primary microglia to recombinant human sTREM2 in vitro increased survival of both [193, 195]. A protective effect has also been reported in vivo after brain injection of sTREM2 into 5xFAD mice [194]. Recently, Bullock et al*.* generated two antibodies against TREM2 extracellular IgV domain that do not affect the putative ligand binding sites [196]. Single-chain variable fragments (scFvs) of these antibodies bound to TREM2, inducing internalization and blocking TREM2 cleavage. These antibodies will be helpful in further dissecting the impact of TREM2 cleavage and sTREM2. Independent of the pathogenic involvement, sTREM2 is becoming a useful marker of AD pathology and cognitive decline. CSF levels of sTREM2 in AD patients positively correlated with the levels of the neurodegenerative markers Aβ42 and T-tau/P-tau in the CSF indicating co-occurrence of AD pathology and microglia activation [197–200]. Higher ratio of sTREM2 versus p-Tau concentrations in the CSF were associated with slower cognitive decline; additionally, high CSF sTREM2 was associated with attenuated amyloid and Tau positron emission tomography [201, 202]. Together, these results suggest that microglia activation slows down AD progression.

## Conclusions

Given the abundance of genetic studies implying microglia in AD, various microglia-targeting immunotherapeutic approaches have been explored aimed at promoting phagocytotic clearance, improving metabolic fitness and debilitating inflammatory response. Pharmacologic depletion of microglia before AD onset and/or during disease progression reduces neuroinflammation and rescues synaptic loss despite persistence of plaques [72, 203, 204]. These studies are consistent with a detrimental function of microglia in the progression of Aβ and tau pathogenesis [70, 72, 186, 203–206]. By contrast, a recent study led by Kiani Shabestari et al. showed that genetic ablation of microglia via deletion of the CSF1R enhancer FIRE induces a shift from parenchymal amyloid plaques to cerebral amyloid angiopathy in 5xFAD mice, which is accompanied by brain calcification and hemorrhages [207]. Considering that microglia play broad roles in CNS development and maintenance, microglial ablation maybe a double-edged sword in AD treatment. Alternative strategies to boost a protective mechanism of microglia using specific monoclonal antibodies are emerging as a promising direction in AD immunotherapy, such as anti-Aβ monoclonal antibody that boost antibody-dependent cellular phagocytosis (ADCP) of opsonized Aβ by microglia [82]. Remarkably, it was recently announced that a phase 3 clinical trial with Lecanemab (BAN2401), a humanized anti-Aβ IgG1 antibody, slowed down the rate of cognitive decline by 25% over 18 months, and the incidence of the brain edema was one-third of that seen with Aducanumab (BIIB037) [208, 209].

As genetic variants linked to reduced TREM2 activity are associated with high risk for human AD [123], TREM2 represents a promising therapeutic target for AD that can be triggered using agonistic antibodies [82, 83, 152]. Anti-TREM2 AL002 [82] is currently tested in a phase 2 clinical trial (ClinicalTrials.gov.: NCT04592874). One challenge with antibody-based therapies is the poor permeability of the blood brain barrier for antibodies. Therefore, engineered antibodies hold great promise to treat AD. A bispecific antibody consisting of a tetravalent TREM2-targeting antibody and an antibody targeting the mouse transferrin receptor (TREM2/αTfR bispecific Ab) has been developed, which strongly facilitates brain delivery and increases microglial TREM2 activation [210]. It is noteworthy that contrasting effects of TREM2 activation have been reported in different AD pathologies and disease stages, suggesting TREM2 activation may be beneficial in a certain time window and in Aβ pathology rather than tau pathology. Novel animal models that recapitulate key phenotype features found in human AD should be considered in developing TREM2-directed therapeutics. Currently, pure amyloid mouse models cause an exacerbated phenotype that does not faithfully represent human AD. Furthermore, pure tauopathy mouse models do not recapitulate the amyloid cascade hypothesis. Perhaps mouse models that are aged and genetically manipulated to progressively develop Aβ plaques and tau tangles may provide better models for testing TREM2 therapeutics. Additional microglial activating receptors have also been pointed out by genetic and experimental studies as new drug candidates, such as TAM receptors and CLEC7A [158, 211, 212]. The development of all these approaches will help to design effective and individually tailored therapeutics for AD.

## Data Availability

Not applicable.

## Abbreviations

AD: Alzheimer’s disease
Aβ: Amyloid beta
NFT: Neurofibrillary tangles
TREM2: Triggering receptor expressed on myeloid cells 2
APOE: Apolipoprotein E
WM: White matter
GM: Gray matter
IGF-1: Insulin-like growth factor 1
DAP12: DNAX activating protein of 12 kDa
PET: Positron emission tomography
CSF: Cerebrospinal fluid
TLR: Toll like receptor
NLRP3: Pyrin (PYD)-domain-containing protein 3
ASC: Apoptosis-associated speck-like protein containing a CARD
TNFα: Tumor necrosis factor α
IFNγ: Interferon gamma
MEF2A: Myocyte enhancer factor 2A
SMAD3: Mothers against decapentaplegic homolog 3
MAFB: Transcription factor MafB
IRF8: Interferon regulatory factor 8
SALL1: Spalt-like transcription factor 1
TGF-β: Transforming growth factor-β
CSF1: Colony-stimulating factor-1
ALS: Amyotrophic lateral sclerosis
MS: Multiple sclerosis
MGnD: Microglial neurodegenerative phenotype
DAM: Disease-associated microglia
IFN-R: IFN-I-response
MHC II: MHC class II
ARMs: Activated response microglia
TRMs: Transiting response microglia
IRMs: Interferon response microglia
CPMs: Cycling/proliferating microglia
WT: Wild type
KO: Knock out
KI: Knock in
KD: Knock down
EOAD: Early-onset Alzheimer’s disease
LOAD: Late-onset Alzheimer’s disease
GWAS: Genome-wide association studies
NHD: Nasu-Hakola disease
LOF: Loss-of-function
GOF: Gain-of-function
CV: Common variant
ERK1/2: Extracellular signal regulated kinase 1/2
PS: Phosphatidylserine
TCA: Tricarboxylic acid
LDs: Lipid droplets
CE: Cholesteryl esters
LDAM: Lipid droplet accumulating microglia
iPSC: Induced pluripotent stem cell
iMG: iPSC-derived microglia-like cell
BMDM: Bone marrow-derived macrophages
PLCγ2: Phospholipase C gamma2
xMGs: Xenografted microglia
ADCP: Antibody-dependent cellular phagocytosis
CNS: Central nervous system
